# Lobster processing by-products as valuable bioresource of marine functional ingredients, nutraceuticals, and pharmaceuticals

**DOI:** 10.1186/s40643-017-0157-5

**Published:** 2017-06-22

**Authors:** Trung T. Nguyen, Andrew R. Barber, Kendall Corbin, Wei Zhang

**Affiliations:** 1 Centre for Marine Bioproducts Development, Flinders University, Adelaide, Australia; 2 Department of Medical Biotechnology, School of Medicine, Flinders University, Adelaide, Australia; 3 Department of Food Science and Technology, Agricultural and Natural Resources Faculty, An Giang University, Long Xuyen, Vietnam; 4 Centre for NanoScale Science Technology (CNST), Chemical and Physical Sciences, Flinders University, Adelaide, Australia

**Keywords:** Lobster processing by-products, Marine functional ingredients and nutraceuticals, Chitin and chitosan, Astaxanthin, Lobster flavors, Lobster lipids, Lobster protein

## Abstract

The worldwide annual production of lobster was 165,367 tons valued over $3.32 billion in 2004, but this figure rose up to 304,000 tons in 2012. Over half the volume of the worldwide lobster production has been processed to meet the rising global demand in diversified lobster products. Lobster processing generates a large amount of by-products (heads, shells, livers, and eggs) which account for 50–70% of the starting material. Continued production of these lobster processing by-products (LPBs) without corresponding process development for efficient utilization has led to disposal issues associated with costs and pollutions. This review presents the promising opportunities to maximize the utilization of LPBs by economic recovery of their valuable components to produce high value-added products. More than 50,000 tons of LPBs are globally generated, which costs lobster processing companies upward of about $7.5 million/year for disposal. This not only presents financial and environmental burdens to the lobster processors but also wastes a valuable bioresource. LPBs are rich in a range of high-value compounds such as proteins, chitin, lipids, minerals, and pigments. Extracts recovered from LPBs have been demonstrated to possess several functionalities and bioactivities, which are useful for numerous applications in water treatment, agriculture, food, nutraceutical, pharmaceutical products, and biomedicine. Although LPBs have been studied for recovery of valuable components, utilization of these materials for the large-scale production is still very limited. Extraction of lobster components using microwave, ultrasonic, and supercritical fluid extraction were found to be promising techniques that could be used for large-scale production. LPBs are rich in high-value compounds that are currently being underutilized. These compounds can be extracted for being used as functional ingredients, nutraceuticals, and pharmaceuticals in a wide range of commercial applications. The efficient utilization of LPBs would not only generate significant economic benefits but also reduce the problems of waste management associated with the lobster industry. This comprehensive review highlights the availability of the global LPBs, the key components in LPBs and their current applications, the limitations to the extraction techniques used, and the suggested emerging techniques which may be promising on an industrial scale for the maximized utilization of LPBs.

**Graphical abstract:**
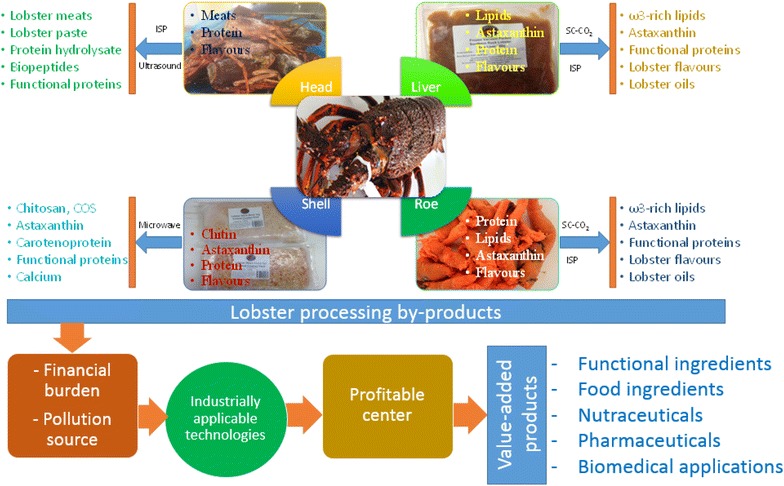
Lobster processing by-product as bioresource of several functional and bioactive compounds used in various value-added products

Lobster processing by-product as bioresource of several functional and bioactive compounds used in various value-added products

## Global lobster processing industry generates a large amount of by-products

**Fig. 1:**
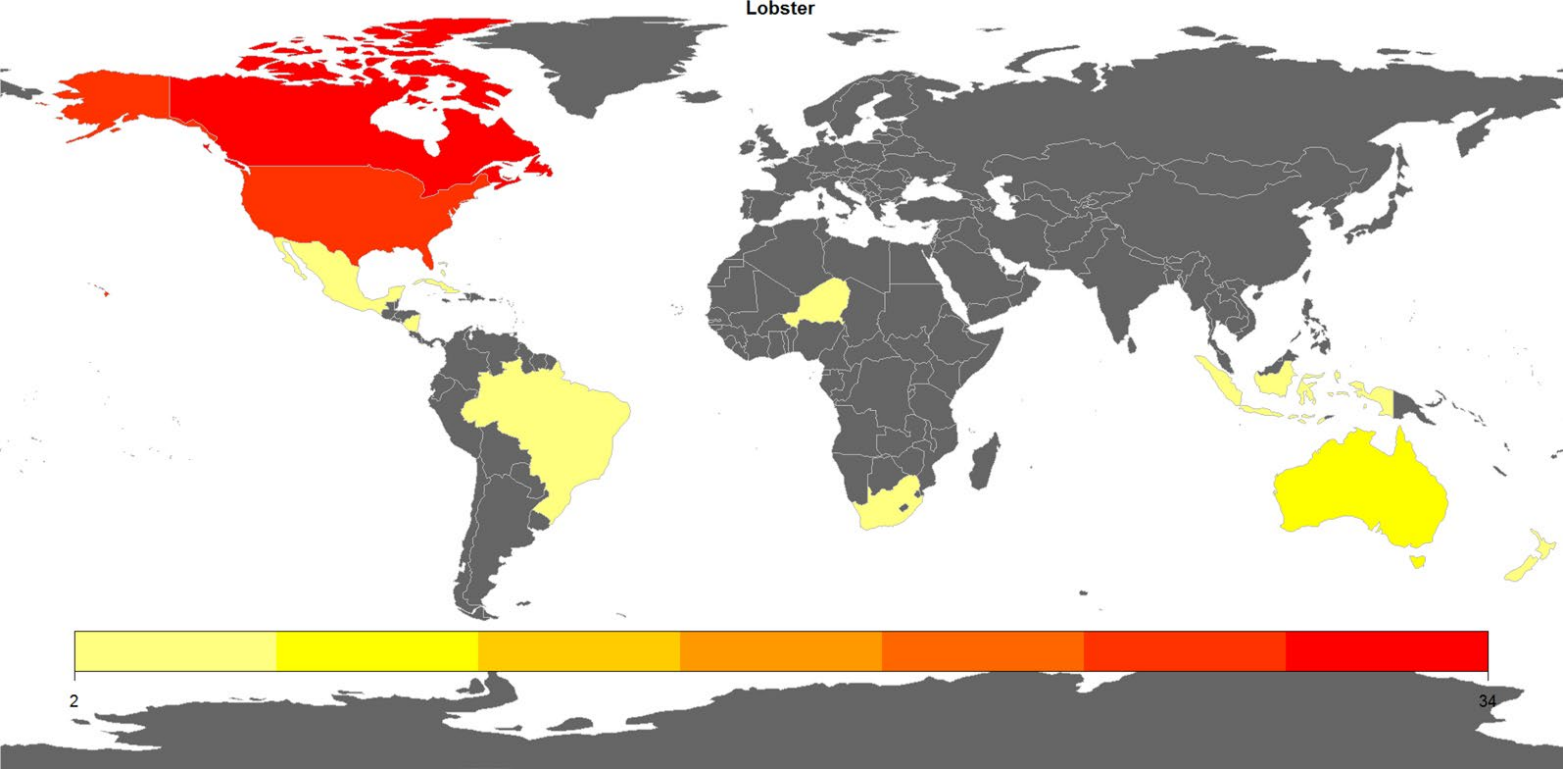
Major lobster-producing countries in the world with their contribution to the global production

In 2004, the global production of lobster yielded 165,367 tons (Holmyard and Franz 2006) which had an estimated value of $3.32 billion. Over the last decade, these figures have been rising to reach 304,000 tons (captures and aquaculture) in 2012 (Sabatini 2015). Lobster production can be found across the world; however, the majority of production is concentrated in only three countries: Canada (34%), America (29%), and Australia (11%) (Fig. 1) (Annie and McCarron 2006). The four main commercial lobster species produced are the American lobster (*Homarus americanus*), Tropical or Spiny lobster (*Panulirus sp*), Rock lobster (*Jasus sp*), and European lobster (*Homarus gammarus*).

**Fig. 2:**
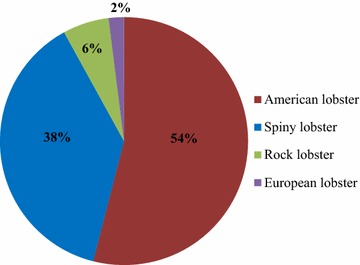
The four main commercial lobster species in the world

The most abundant species produced in the world is the American lobster which is mainly harvested in Canada and America (Fig. 2) (Annie and McCarron 2006). In 2012, both Canada and America produced 140,000 tons of lobsters (Thériault et al. 2013) with the majority (74,790 tons, valued at $662.8 million) originating from Canada (Ilangumaran 2014). The second-most readily available commercial species is Spiny lobster accounting for 38% of the global production, while the contribution of the Rock lobster is 6%. This latter species is predominantly harvested from Australia, which includes four main commercial species: Western rock lobster (60%), Southern rock lobster (30%), Tropical rock lobster (8%), and eastern rock lobster (2%) with the total yield about 9650 tons annually (Gary 2012). The term ‘Rock lobster’ has been used to describe lobster species such as *Jasus* and *Panulirus* which are caught by Australian lobster fishery (Holmyard and Franz 2006).

As lobster are consumed globally but are predominantly produced in a few countries, there is a rapidly growing export market of lobsters. Although live lobsters are preferred by consumers around the world, the export of live lobsters is limited due to its high cost, complexity, and high rates of mortality and loss during shipment. In contrast, processed lobsters has several advantages such as ease of handling in transport and storage, extended shelf life, availability of the products, convenience in food preparation, and higher potential to adding value to raw products. This ease in handling and increase in profits have resulted in over half of the landed lobster in the major lobster-producing countries being processed (Barker and Rossbach 2013; Denise and Jason 2012; Ilangumaran 2014).

**Fig. 3:**
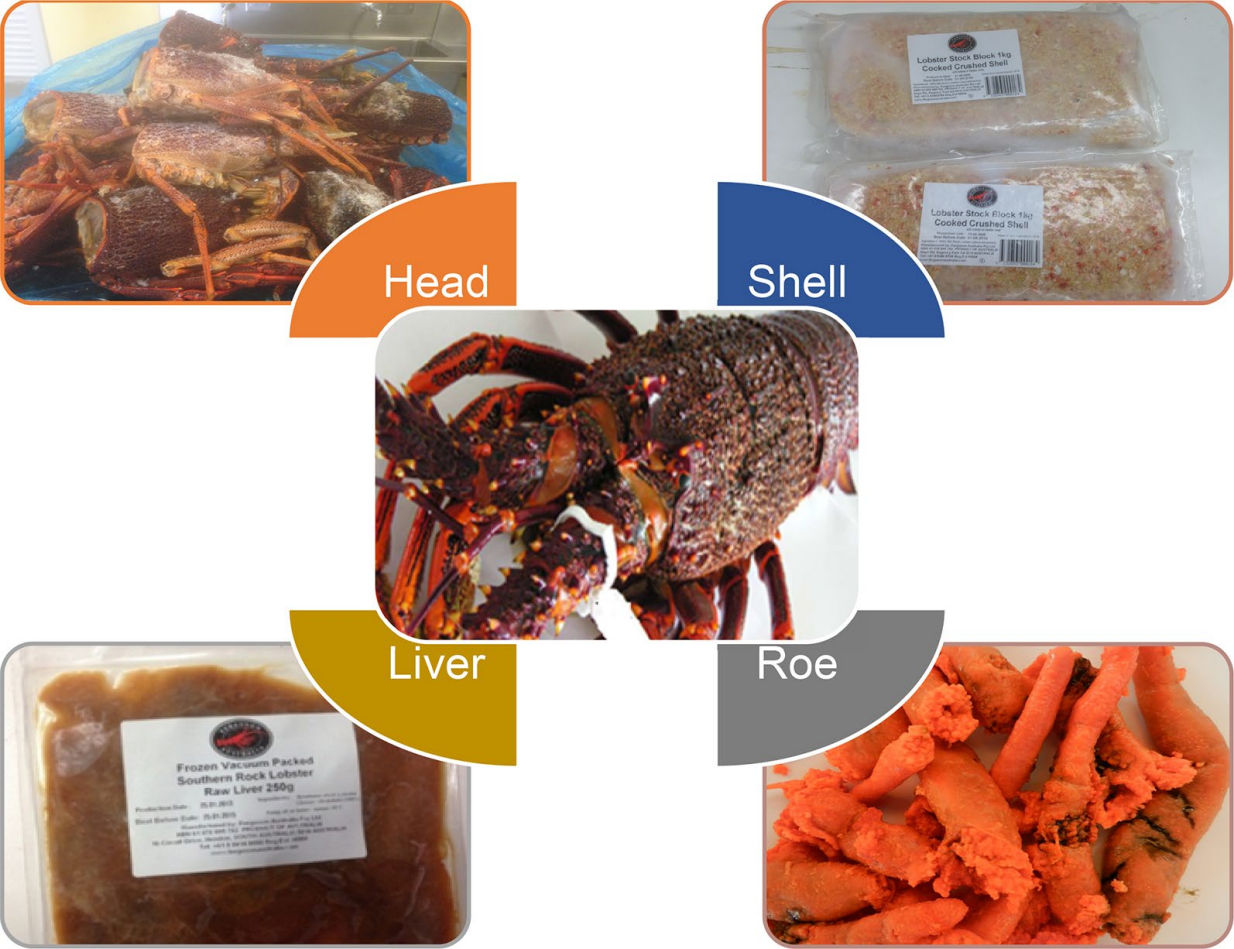
Different by-products (heads, livers, shells, and eggs) generated from the commercial lobster processing industry

Lobsters are commercially processed into various products such as fresh lobster meat, picked lobster meat, canned lobster, lobster medallion, whole cooked lobsters, and frozen lobsters (Holmyard and Franz 2006). During processing, the inedible parts are removed including heads, shells, roe, and livers (Fig. 3), and are traditionally discarded. The types and proportion of lobster processing by-products (LPBs) generated vary depending on the processing process but on average accounts for around 75% (w/w) of the starting material (Table 1). As a result of this, the annual estimate of LPBs produced from the major lobster processing countries (Canada, America, and Australia) is about 50,000 tons.

**Table 1:** The amount of by-products generated from the different lobster processing industries

Lobster processing industries	Types of by-products	Percentage of by-products based on starting material (%)	References
Canning of Canadian lobsters	Lobster body	45	Ross (1927)
Canadian lobster meat	Lobster head, hard carapace, viscera, mandibles, and gills	>75	Tu (1991)
Brazilian lobster tails	Lobster head (cephalothorax)	75	Vieira et al. (1995)
Fresh meat picked from Australian rock lobster	Lobster head, shell, and viscera	60	Lien (2004)
High hydrostatic pressure production of American lobster meat	Lobster shells, viscera, residual meat	75–80	Denise and Jason (2012)

To maximize the production yield and profit, lobster processors have begun to utilize some LPBs to produce several products such as lobster tomalley, lobster roe, lobster concentrate, and lobster meat paste (Holmyard and Franz 2006). However, the amount of LPBs being utilized compared to the tons generated is still very limited. The slow uptake and growth of industries using this waste material may be attributed to the current lack of efficient and standardized techniques to transform these materials into a marketable form. Thus, the vast majority of LPBs is discarded at a cost incurred by lobster processors. In some countries such as Australia, this fee can be in excess of $150 per ton (Knuckey 2004; Yan and Chen 2015). In addition, not only is the disposal of lobster by-products and waste management a financial burden for lobster processing companies globally costing an estimated $7.5 million per year, but is also considered to be environmentally unfriendly due to dumping in the landfill or the sea (Chen et al. 2016; Yan and Chen 2015). This could create disposal problems and environmental pollutions (Hamed et al. 2016; Sayari et al. 2016). Moreover, this underutilizes a marine bioresource that could be mined to produce several valuable ingredients for a wide range of commercial industries. Shell waste, for instance, has been considered as a source of useful chemicals for many commercial applications (Chen et al. 2016; Yan and Chen 2015). Crude crustacean shells or chitin derived from chitin shells could be used for production of low molecular weight chitosan and chitin oligomers with numerous applications employing simple processes (Chen et al. 2017; Zhang and Yan 2016). As a result, the utilization of these LPBs for recovery of marine functional ingredients, nutraceuticals, and pharmaceuticals for incorporation into highly value-added products could bring significantly both economic and environmental benefits to regions where lobsters are processed.

## LPBs containing several valuable components

### Proteins

By-products generated from lobster processing industry are protein-rich sources for recovery. For example, the lobster liver (green) contains up to 41% protein on a dry basis (Nguyen et al. 2015), while the lobster head containing residual meats (body, breast, and leg) up to 20% of the lobster weight (Vieira et al. 1995) would be another major high-quality protein source. In addition, lobster shells (carapace) constituted by a large amount of proteins (about 25%) are also another potential source of protein for mining (Nguyen et al. 2016).

The amino acid profile of crustacean protein such as lobster is comparable to that of red meat protein, but it contains more nonprotein nitrogen (amino acids, small peptides, trimethylamine oxide (TMAO), trimethylamine, creatine, creatinine, and nucleotides) ranging from 10–40%. Thus, crustacean protein is more palatable than meat proteins (Venugopal 2009). As compared with other marine species such as finfish, proteins derived from crustacean generally contain larger amounts of arginine, glutamic acid, glycine, and alanine; this makes crustacean proteins more palatable than finfish proteins. Due to its ideal essential amino acid pattern, moreover, the nutritional value of crustacean protein is equal to or better than that of milk protein (casein) and red meat proteins (Venugopal 2009) or soya-bean proteins (Yan and Chen 2015). Lobster proteins are rich in all the essential amino acids (EAAs) with its proportion approximately reaching 41.2% for protein in lobster head meat (Vieira et al. 1995) and 34% for lobster shell protein (Nguyen et al. 2016). Especially, the nutritional value of lobster protein is fortified significantly by its natural combination with a large amount of astaxanthin (295 μg/g) as a powerful antioxidant to form a protein complex known as carotenoprotein. This protein was found in lobster shells with high proportion (16%) (Tu et al. 1991).

Apart from its high delicacy, palatability, and nutritional values, lobster proteins have also been shown to have excellent functional properties. For example, protein derived from lobster head meats has been shown to have excellent wettability, high solubility, and emulsification (Vieira et al. 1995). Solubility of lobster shell protein (LSP) recovered either by aqueous extraction or enzymatic digestion was over 93%, which is independent of the pH value and the ionic strength of the solution used (Nguyen et al. 2016; Oviedo et al. 1982). High water binding of LSP was also reported by the fact that beef mince when added with 2% of LSP resulted in its water-binding capacity being 2.5 times higher than that when added with egg white protein (Nguyen et al. 2016). In addition, lobster protein hydrolysate (LPH) performed an excellent emulsifying property (69.7 vs 50.3 m^2^/g of cow gelatin) (He et al. 2016).

### Chitin and chitin derivatives

**Fig. 4:**
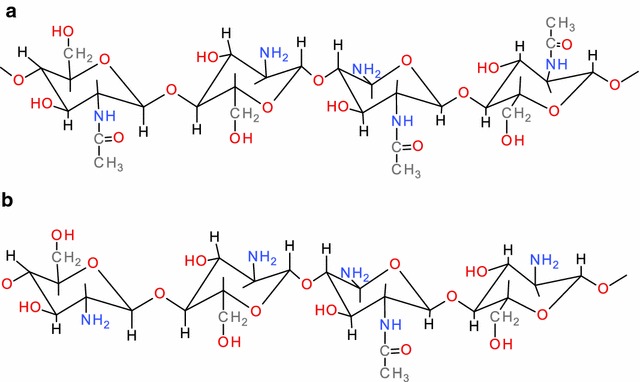
The chemical structure of the main polymer present in lobster shell: chitin (**a**), and the *N*-deacetylation form of chitin, chitosan (**b**)

Chitin is a cationic linear polysaccharide composed of β-(1–4)-linked *N*-acetyl-d-glucosamine monomers (Fig. 4a) and is the second-most abundant biopolymer only after cellulose in the biosphere (Kumar 2000; Kurita 2006). Chitin is present in lobster shells with contents of 16–23% in the form of α-chitin (Lien 2004; Rinaudo 2006; Tu 1991). Chito san represents a family of *N*-deacetylated chitin with various degrees of deacetylation (Fig. 4b), while chito-oligosaccharides (COS) are derived from chitin and chitosan by either chemical or enzymatic hydrolysis.

LPBs such as lobster shell waste are abundant and rich in chitin attributing to its importance as a potential source of commercial chitin. The utilization of LPBs for recovery of chitin has received a great interest since chitin is a natural biopolymer with high biodegradability, biocompatibility, and nontoxicity (Karagozlu and Kim 2015). Derivatives generated from chitin such as chitosan and COS have significant potential economic values with more than 200 applications in water treatment, food, agriculture, healthcare products, environmental sector, pharmaceuticals, and biomedicine (Kaur and Dhillon 2013; Muzzarelli 1989; Sandford 1989; Synowiecki and Al-Khateeb 1997). There is an increasing global demand for chitin and chitin derivatives with the estimated quantities of 11,400 tons for chitin and 33,400 ton for chitin derivatives (Hayes 2012). Economic value of chitin and chitin derivatives was $2.0 billion in 2016, but this figure is estimated to increase significantly to $4.2 billion by 2021 (Pathak 2017).

### Lipids

Lipid is another valuable constituent of LPBs due to its nutraceutical-rich composition (polyunsaturated fatty acids (PUFAs); ω-3 fatty acids; and lipid-soluble vitamins). Lobster body contains less than 2% of lipids (Shahidi 2006), but concentration of this constituent is significantly higher in some other parts of the lobsters. Cephalothorax, a by-product generated from lobster processing industry, is one of the lipid-rich parts of lobster with various lipid contents depending on lobster habitat, season, and species. In summer season, Norway lobster cephalothorax has the highest lipid content with a value of 11.5% (Albalat et al. 2016). Cephalothorax of Norway lobster contains lower lipid content than that of Australian lobster which accounts for up to 19.4% (Tsvetnenko et al. 1996). Lobster liver is another lipid-rich part of lobster with the lipid content of which is up to 24.3% (Nguyen et al. 2015). Among crustaceans, LPBs contain more lipids than crab and shrimp by-products, while this value is comparable to that of krill being used for lipid production (Albalat et al. 2016). Lobster lipids are rich in PUFAs and ω-3 fatty acids as reported in the study of Tsvetnenko et al. (1996): lipids extracted from lobster cephalothorax contained 22.6% of fatty acids in which ω-3 fatty acids accounted for 50.8%. However, the amounts of PUFAs and ω-3 fatty acids were significantly higher (22.6 vs 31.3%, 50.8 vs 58%) when lipids were recovered from lobster livers using supercritical carbon dioxide (SC-CO_2_) extraction technique (Nguyen et al. 2015). Richness of PUFAs and ω-3 fatty acids in lobster lipids is comparable to that of menhaden lipids (Tsvetnenko et al. 1996) or even higher than that of krill lipids (Albalat et al. 2016). Particularly, lobster lipids also contain carotenoids and astaxanthin with concentrations of 70.4 and 41.6 μg/mL, respectively (Nguyen 2017).

### Astaxanthin

Astaxanthin is the oxygenated derivatives of carotenoids occurring widely and naturally in marine organisms including crustaceans (lobster, shrimp, crab, and krill) and fish (salmon, sea bream). Astaxanthin is one of the first pigments isolated and characterized from lobster (Kuhn and Soerensen 1938). By-products generated from lobsters, shrimps, crabs, crayfish, and krill are a vital source of natural carotenoids, mainly astaxanthin (Sachindra et al. 2007). Apart from containing a large proportion of mineral salts (15–35%), proteins (25–50%), chitin (25–35%), and lipids (19.4–24.3%) (Lee and Peniston 1982; Nguyen et al. 2015), LPBs are also constitutive of a certain amount of carotenoids. Astaxanthin content in crustaceans varies depending on species, season, and environmental grown conditions, but lobster by-products contain double the amount of astaxanthin compared with shrimp (Table 2). Astaxanthin exists in a free form and/or in a complex form known as carotenoprotein. Due to their containing a high proportion of astaxanthin, LPBs have been utilized for the recovery of astaxanthin (Auerswald and Gäde 2008; Gäde and Auerswald 2005). Two processes were patented for astaxanthin extraction from lobster heads (Kozo 1997; Sunda et al. 2012), while food-grade astaxanthin was recovered from the shells of American lobster generated from the high hydrostatic pressure process (Denise and Jason 2012).

**Table 2:** Total astaxanthin in by-products of lobsters compared with other crustacean species

Source	Total astaxanthin (mg/100 g)	References
Shrimp (*P. borealis*)	4.97	Torrissen et al. (1981)
Crawfish (*P. clarkii*)	15.30	Meyers and Bligh (1981)
Backs snow crab (*Ch. opilio*)	11.96	Shahidi and Synowiecki (1991)
Lobster (*Homarus americanus*)	9.80	Tu (1991)

## Various applications of extracts derived from LPBs

The most common traditional utilization of LPBs is their use as a source of nutrients for soil amendment considered as an informal way of disposal (Cousins 1997), but it brings no economic benefits for lobster producers. Recently, LPBs have been studied as an important bioresource in the recovery of marine functional ingredients, nutraceuticals, and pharmaceuticals for numerous applications (Table 3).

**Table 3:** The different types of lobster by-products generated with their valuable components for potential areas of applications

Lobster by-products	Functional ingredients, nutraceuticals, pharmaceuticals	Suggested application areas	References
Lobster shells (carapace)	Chitin, chitosan	Water treatment	Gustavo et al. (2005); Pathiraja (2014)
	Chitin, chitosan, chitin-oligosaccharides, chitosan-oligosaccharide	Agriculture	Borges et al. (2000); Falcón et al. (2002); Cabrera and Van Cutsem (2005); Falcón Rodríguez et al. (2010); Ilangumaran (2014)
	Chitosan, chitosan film	Food processing and preservative	Defang et al. (2001); Garcıa et al. (2015)
	Water-soluble chitosan, chitosan particles	Pharmacy	Safitri et al. (2014); De la Paz et al. (2015)
	Chitosan film	Biomedicine	Malho et al. (2014); Qi (2015)
	Carotenoprotein	Aquafeed	Dauphin (1991); Simpson et al. (1993); Tu (1991); Tu et al. (1991)
	Astaxanthin	Food, nutraceutical, pharmaceutical; feed additive	Auerswald and Gäde (2008); Denise and Jason (2012); Gäde and Auerswald (2005)
	Proteins	Food and nutraceutical	Nguyen et al. (2016); Oviedo et al. (1982)
	Flavors and nutrient broth	Crackers, biscuits	Lien (2004)
Lobster heads (cephalothorax)	Body meat, breast meat, and leg meat	Lobster paste, canned products	Ross (1927)
	Lobster meat	Gourmet food products	Meyers and Machada (1978)
		Feed additive	Daniel (2008)
	Lobster protein hydrolysate	Flavor enhancer, protein supplement	Vieira et al. (1995)
Lobster roes	Raw roe	Lobster paste, canned products	Ross (1927)
Lobster livers (hepatopancreas)	Raw liver	Lobster paste, canned product	Ross (1927)
	ω-3 rich lipids	Lobster oils, infused oils	Nguyen et al. (2015); Tsvetnenko et al. (1996)
Lobster blood (hemolymph)	Phenol oxidase	Anti-microbial proteins	Fredrick and Ravichandran (2012)
	Crustin	Anti-microbial proteins	Battison et al. (2008); Pisuttharachai et al. (2009)
	Bioactive fragment	Pharmaceutical and/or cosmetic treatment of viral and other neoplastic or pre-neoplastic mammalian tissue lesions	Bayer (2015)

### Lobster protein: dietary protein supplement, food functional ingredients, or flavorings

The recovery of edible meat from lobster by-products is not novel. However, the use of these by-products as a source of sustainable proteins for food products has been less explored. In one study, the meats (body meat, breast meat, leg meat, roe, and liver) recovered from Canadian lobster by-products were used to create a canned food product and lobster paste (Ross 1927). Although the final products prepared from these protein sources were flavor-rich, nutritious, and palatable, the feasibility for commercialization was found impractical as the methods used for residual meat recovery were inefficient. To address this key problem, a process was developed for the recovery of food-grade lobster meats from spiny lobster head by-product by freezing the heads and later cutting them for meat picking. The recovered lobster meat was sold as high-value, gourmet food products (Meyers and Machada 1978).

The actual flavor of lobster itself is considered a highly valued product, which may be extracted and sold, creating an additional processing stream for lobster wastes. The practice of converting LPBs into natural lobster flavors has been standardized and has now become an established industrial practice. The cephalothorax of Brazilian lobster by-products (*Panulirus* spp.) has been utilized for lobster flavor production by enzymatic hydrolysis of lobster head meats (Vieira et al. 1995). Hydrolyzed lobster protein could be used as flavor enhancers for various formulated food products. The key aromatic components derived from cooked tail meat of American lobster (*Homarus americanus*) was investigated by Lee et al. (2001). In this study, 3-methylbutanal, 2,3-butanedione, (Z)-heptenal, 3-(methylthio)propanal, 1-octadien-3-one, and (E,Z)-2,6-nonadienal were identified as dominant aroma components of cooked American lobster tail meat with high odor intensities. By this reason, flavorants extracted from lobster shells by either frying with edible oils or cooking for nutrient extraction were used for production of infused lobster oil and lobster cracker biscuit (Lien 2004). Due to possessing several functional properties that are favorable for use in food industry, LSP has also been characterized and trialed for various applications including food-functional ingredients or as protein supplements (Nguyen et al. 2016; Oviedo et al. 1982), enhancing water-binding or reducing lipidemic effects of meat protein (Nguyen et al. 2016), emulsifier (He et al. 2016).

### Lobsters chitin, chitosan, and their derivatives as natural biopolymers for multiple applications

**Fig. 5:**
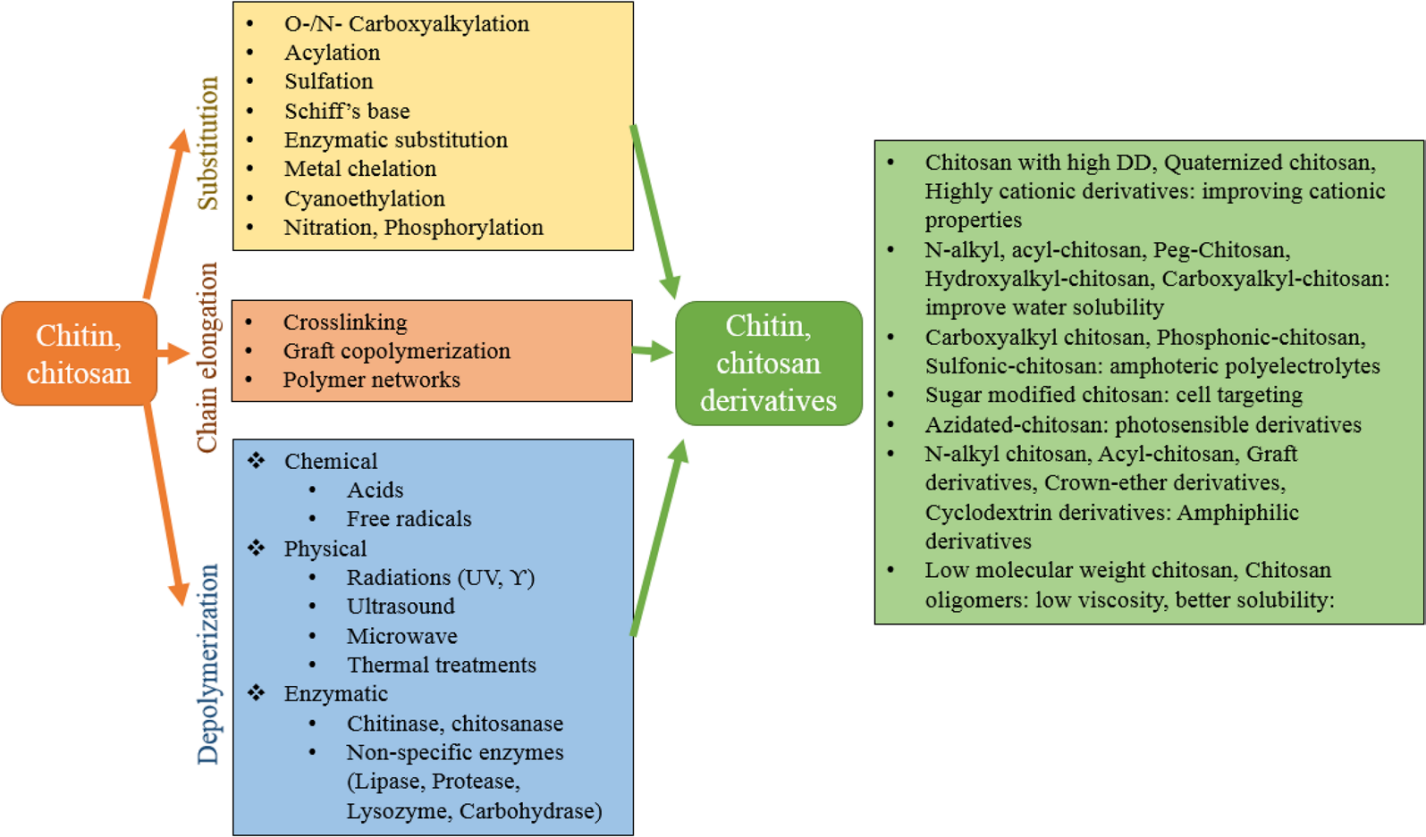
The methods used to produce chitin/chitosan derivatives with the improved functional properties

In recent decades, a greater knowledge of chitin chemistry accompanied by the increased availability of crustacean shells as by-products of the crustacean-processing industry have led to significant development and wide applications of chitin and its derivatives produced by several pathways (Fig. 5). In particular, derivatives from chitin such as chitosan and COS are highly valued compounds as they have more than 200 commercial applications (Kaur and Dhillon 2013; Muzzarelli 1989; Sandford 1989; Synowiecki and Al-Khateeb 1997). In addition, the biopolymers generated from marine crustaceans, lobster chitin, chitosan, and their derivatives have been used in multitude of industries including water treatment, agriculture, food production, pharmaceuticals, and biomedicine (Table 4).

**Table 4:** Applications of lobster chitin, chitosan, and their derivatives

Industry	Applications	References
Water treatment	Removal of mercuric ion	Peniche‐Covas et al. (1992)
	Removal of reactive dyes from aqueous solution	Juang et al. (1996, 1997)
	Removal of heavy metals (Cu, Hg)	Gustavo et al. (2005)
	Dye absorbents for treatment of industrial effluent	Pathiraja (2014)
Agriculture	Seedling growth and antimycorrhizal in tomato crop	Iglesias et al. (1994)
	Fungicides on plant fungal diseases	Pombo (1995)
	Amending media and seeds for inhibition of pathogen fungus growth	Borges et al. (2000)
	Inducing systemic resistance agents in tobacco plants	Falcón et al. (2002)
	Elicitors of plants defense reactions	Cabrera and Van Cutsem (2005)
	Plant growth regulators	Hipulan (2005)
	Inducing defensive agents	Cabrera et al. (2010); Falcón Rodríguez et al. (2010)
	Plant protection	Ilangumaran (2014)
Food	Organic polymer flocculants	Defang et al. (2001)
	Edible or biodegradable films	Casariego et al. (2008, 2009)
	Biodegradable packages	Hudson et al. (2015)
	Fiber and nutrient supplement	Harikrishnan et al. (2012)
	Antioxidants, antimicrobials	Garcıa et al. (2015); Sayari et al. (2016)
Pharmaceuticals	Direct compression excipients for pharmaceutical application	Mir et al. (2008)
	Co-diluent in direct compression of tablets	Mir et al. (2010)
	Water-soluble lobster chitosan salt as materials for drug carriers	Cervera et al. (2011)
	Targeted drug delivery film	Bamgbose et al. (2012)
	Bio-mouth spray for anti-halitosis	Safitri et al. (2014)
	The stability and safety of lobster chitosan salts	De la Paz et al. (2015); Lagarto et al. (2015)
	Natural additive for pharmaceuticals	Sayari et al. (2016)
Biomedicine	Immobilization of multi-enzyme extract	Osinga et al. (1999)
	Novel hybrid biomaterials for medical application	Malho et al. (2014)
	Biomimetic functional materials or epithelial treatment	Qi (2015); Da Silveira et al. (2013)

### Water treatment

One of common applications of chitin and its derivatives is for the treatment of water. Chitin and chitosan with their high absorbance, chelating, and affinity properties have been used as coagulation agents, chelating polymers, or bio-absorbents in water treatment for decades. In particular, chitin and chitosan prepared from lobster have shown high affinity for metal chelating. Peniche‐Covas et al. (1992) reported lobster chitosan could effectively remove mercuric ions from solutions. Lobster chitosan was later used successfully for separation of heavy metals (Cu, Hg) (Gustavo et al. 2005). The heavy metal removal efficiency of lobster chitosan was comparable to that of commercial resin. Lobster chitin/chitosan has been used for the removal of reactive dyes (vinyl sulfone and chlorotriazine) from aqueous solution (Juang et al. 1997). Lobster chitin/chitosan produced from this study had significantly high adsorption capacity of reactive dyes (50–500 mg/L) compared with a conventional absorbent (activated carbon). The result of this study has been recently amplified by a study of Pathiraja (2014) where lobster chitin was used as dye absorbents for effective treatment of industrial effluent.

### Agriculture

Chitin and its derivatives have been shown to have profound beneficial effects when used for agriculture applications. For example, these polymers stimulate plant growth and improve crop yields (Badawy and Rabea 2011; Deepmala et al. 2014; El Hadrami et al. 2010; Sharp 2013). In addition, when applied to crops, they exhibit toxicity to plant pests and pathogens, induce plant defenses, have high anti-fungal activity, and stimulate the growth and activity of beneficial microbes (Pombo 1995; Sharp 2013). Based on these findings, cheap sources rich in chitin such as lobster shells have been trialed as fungicides, biocides, and bio-stimulants.

Other biological effects induced by lobster chitosan when applied to plant seeds were reported by Borges et al. (2000). Those authors used chitosan prepared from lobster chitin to amend agar and coat tomato seeds together with menadione sodium bisulfite for inhibition of pathogenic fungus and disease development on tomato seeds. The results showed that lobster chitosan significantly inhibited the fungal growth and diminished disease occurrence in the roots from seeds, which had been treated. In another study, lobster chitin was shown to have positive effects on seedling growth and mycorrhizal infection of tomato crop (Iglesias et al. 1994). Furthermore, chitin extracted from Cuban lobster shells has been shown to have biological functions. Researchers at the University of Havana prepared chitosan from lobster chitin to coat tomato seeds as well as encapsulate somatic embryos to produce artificial seeds for accelerating yields. Under laboratory conditions, the coated seeds exhibited considerably higher rates of germination and growth compared with the noncoated seeds. From this study, it was concluded that the lobster-shell-derived chitosan served as a biological stimulant yielding better seed germination, increased plant height, enhanced stem thickness, and dry biomass yield (Hareyan 2007).

Chitin, chitosan, and COS recovered from lobster shells have also been studied for their biocide and biostimulant activities for agricultural applications. A study carried out by Falcón et al. (2002) investigated the use of lobster chitosan and chitosan oligomers for crop protection. Both lobster chitosan and its enzymatic hydrolysate exhibited high pathogen-resistant activity against *Phytophthora parasitica* on tobacco plants at low concentrations ranging from 5–500 mg/L. With this success, the study was extended by preparing various chitosan and chitosan-oligomers from Cuban lobster chitin. Both lobster chitosan and its derivatives were tested for antimicrobial activity (versus fungus and oomycetes) and their ability to induce defensive and protective responses in tobacco and rice plants. Some of the lobster chitosan derivatives were found to be active protectants against infection for both cultivars at field scale (Falcón Rodríguez et al. 2010).

In another study, Peters et al. (2006) used raw lobster shells and compost as a soil amendment to supply nutrients for plants and as a biological control method for soil-borne fungi pathogenic to potatoes. The results showed that lobster shells served as a nutrient source for plant growth, enhanced beneficial soil microbial communities, suppressed soil-borne diseases, and was an organic production process. Following this success, Ilangumaran (2014) studied isolation of soil microbes for bioconversion of lobster shells into chitin and chitin-oligomers for plant disease management. The extracted bioproducts showed significant induction of disease resistance in *Arabidopsis* confirming the findings in previous studies.

### Food industry

The use of chitin, chitosan, and their derivatives in food industry has aroused a great interest in recent years because these biopolymers possess several interesting biological activities and functional properties. Chitin recovered from Spiny lobster shells was used to produce low molecular weight chitosan for food applications using gamma irradiation. Lobster chitosan produced from this irradiation process had high antioxidant activity and was proposed to be utilized as a food preservative (Garcıa et al. 2015). Apart from that, chitosan originated from Norway lobster shells had excellent antimicrobial activity against several food-poisoning bacteria and fungus (Sayari et al. 2016). This makes lobster chitosan has a great potential to be used for preservation of foods from microbial deterioration. To recover soluble proteins from food-processing processes, several compounds could be used, but chitosan and chitosan complexes have a great potential (Lu et al. 2011; Wibowo et al. 2007); besides, chitosan prepared from lobster showed promising results for recovery of solids in food-processing plants (Defang et al. 2001). Moreover, chitin and chitosan could be used for producing functional biomaterials (membranes, films, and packages) used for applications in the food industry (e.g., processing, packaging, or storage). Lobster chitosan films were used as the film matrix in combination with clay micro/nanoparticles for the preparation of chitosan/clay films (Casariego et al. 2008, 2009). With its significant improvements in physical properties (water solubility, water vapor, oxygen and carbon dioxide permeability, and optical, mechanical, and thermal properties), the lobster chitosan/clay film was proposed for use in coating in order to extend the shelf life of food products (Casariego et al. 2008, 2009). Apart from retarding moisture migration and the loss of volatile compounds, reducing the respiration rate, and delaying changes in textural properties, another advantage of chitosan films is that it is biodegradable. Thus, chitosan films are environmentally friendly alternatives to synthetic, nonbiodegradable films, which may be further modified to create biodegradable food packages (Hudson et al. 2015).

### Pharmaceuticals and biomedicine

Recently, the applications of chitin, chitosan, and their derivatives in pharmaceuticals and biomedicines have received more attention not only because they are biocompatible, biodegradable, and nontoxic (Jeon and Kim 2000), but also because they exhibit several biological and physiological characteristics with known medical benefits. For example, these polymers and their derivatives are antioxidant, antimicrobial, anticancer, immune-stimulant, hypocholesterolemic, hypoglycemic, angiotensin-I-converting enzyme (ACE) inhibitors, and anticoagulant (Sayari et al. 2016; Wijesekara and Kim 2010).

Apart from the above, chitin and chitin derivatives also possess several physical properties that are favorable for their applications in pharmaceutical and biomedical sectors. Chitin and chitosan prepared from lobster were studied on their deformation and compaction properties for use as pharmaceutical direct compression excipients (Mir et al. 2008). In comparison with other established direct compression excipients (microcrystalline cellulose), lobster chitin/chitosan performed better at both tendencies of plastic deformation and compression behavior. This result indicates that lobster chitin and chitosan have a potential use as co-excipients for direct compression applications.

The most commonly used chitosan derivatives used in drug delivery are the water-soluble lobster chitosan acid salts (Cervera et al. 2011). Lobster chitosan salts prepared by spray-drying have a higher tendency toward sphericity, which are good excipients for pharmaceutical applications. Moreover, lobster chitosan acid salts maintain their physical, chemical, and microbiological characteristics for a period of 12 months when stored correctly at room temperature in a dry place (De la Paz et al. 2015). Apart from being stable for extended periods (1 year), the toxicity levels of lobster chitosan acid salts are negligible. This was indicated by a study investigating the single- and repeated-dose toxicity of chitosan and its salts (lactate and acetate) on rats. At oral doses of 2000 mg/kg, no fatalities or changes in the general behavior of the rats in both the acute- and repeated-dose toxicity studies were observed (Lagarto et al. 2015). This led us to a conclusion that chitosan obtained from lobster shells may be safe for use in the pharmaceutical industry.

The welling potential of lobster chitosan film in various solvents was determined for use as targeted drug delivery or drug film (Bamgbose et al. 2012). Since it can generate a membrane with structure porous and stability in several organic solvents, lobster chitosan film could be incorporated in devices for development of targeted drug delivery. Recently, novel hybrid biomaterials for medical application was also prepared from lobster chitosan (Malho et al. 2014). A bifunctional protein was successfully attached to lobster chitin to generate biosynthetic materials with advanced functional properties. Bio-inspired chitin/protein nanocomposites were developed using lobster chitin nanofibers and recombinant chitin-binding resilin (Qi 2015). Furthermore, chitosan nanoparticles with its antioxidant, antimicrobes in combination with high absorption have been used as pharmaceutical ingredients. Recently, Safitri et al. (2014) reported chitosan nanoparticles produced from lobster had positive results in prevention and treatment of halitosis. Based on these findings, the lobster chitosan nanoparticles were used as an active ingredient in a anti-halitosis bio-mouth spray. Ongoing advances such as these could considerably expand the use of lobster chitin nanofiber-based composites and functional materials.

### Lobster lipids: as source of nutraceuticals, pharmaceuticals, and flavorants

Although lipids have often been condemned, the use of lipid and its products has drawn a dramatic interest in recent years due to findings related to their effects on human health. Apart from enhancing flavor, texture, and mouthfeel to foods, lipids also provide essential fatty acids [eicosapentaenoic acid (EPA), docosahexaenoic acid (DHA), arachidonic acid (AA), and γ-linolenic acid], fat-soluble vitamins (A, D, E, K), and other minor components (phospholipids, tocopherols, tocotrienols, carotenoids, sterols, and phenolic compounds) (Rizliya and Mendis 2014; Shahidi 2006). Particularly, the roles of carotenoids, EPA, and/or DHA in heart health, mental health, brain, and retina development, have been well documented (Alabdulkarim et al. 2012; Guerin et al. 2003; Swanson et al. 2012; Yamashita 2013), and such lipid constituents have been regconized as nutraceuticals and pharmaceuticals for improving human health. By this reason, fish oils have been used for enriching DHA and EPA of many food products such as powder milk formulate, salad oil, fruit beverage, vegetable juice (Kolanowski and Berger 1999), dairy products (Kolanowski and Weißbrodt 2007), soft goat cheese (Hughes et al. 2012), or cookies (Jeyakumari et al. 2016). Lobster oils have a potential use as a source of natural ω-3 fatty acids for many fortified products since they have been demonstrated to contain PUFAs and ω-3 fatty acids as high as that of fish oil (menhaden) or krill oils (Albalat et al. 2016; Tsvetnenko et al. 1996). Moreover, higher bioavailability of fatty acids derived from crustacean oils compared to those of fish oils (Köhler et al. 2015) together with the antioxidant superior of crustacean oils provided by carotenoids make them ideal for application as a novel and beneficial food ingredient (Tetens 2009) or as oil supplement (Köhler et al. 2015). With a significant richness in astaxanthin, PUFAs, and ω-3 fatty acids, lobster oil was suggested for use as a dietary supplement (Nguyen 2017) since oils produced from fish livers are often considered as an important source of vitamins A and D with several therapeutic properties (Gunstone 2006; Rizliya and Mendis 2014). In addition, lobster liver oil contains very strong specific flavors, which combined with the unique and inherent ability of oils for absorbing and preserving flavors, would be a promising application for flavor industry. For this reason, lobster lipids were investigated for production of infused lobster oil, salt plated with lobster flavors, and lobster seasoning, and they obtained promising results (Nguyen 2017).

### Astaxanthin as a powerful antioxidant

Oxidative molecules or free radicals such as hydroxyls, peroxides, and reactive oxygen species generated during normal aerobic metabolism are necessary for sustaining life processes. However, under certain conditions or periods of exposure such as physiological stress, air pollution, smoking, chemical inhalation, or exposure to UV light, the increased production of these free radicals can be detrimental. This threat arises due to the highly reactive nature of free radicals with essential cellular components such as proteins, lipids, carbohydrates, and DNA (Di Mascio et al. 1991). As a result of oxidative damage through a chain reaction known as oxidative stress, proteins and lipids are oxidized, while DNA is severely damaged. It has been suggested that diseases such as macular degeneration, retinopathy, carcinogenesis, arteriosclerosis, and Alzheimer may be induced by such damages (Maher 2000).

The human body controls and reduces oxidation by self-producing enzymatic antioxidants including catalase, peroxidase, super oxide dismutase, and other antioxidant activity molecules. However, the levels of these compounds in many cases are not sufficient to protect the body against oxidative stress, and an additional supplement of water-soluble antioxidants (vitamin C) and lipophilic antioxidants (vitamin E, carotenoids: beta-carotene and astaxanthin) are required. The use of astaxanthin as an antioxidant has been receiving significant attention because it possesses superior antioxidant activity. Antioxidant activity of astaxanthin was found to be 10 times higher than that of zeaxanthin, lutein, canthaxanthin, and β-caroten, and 100 times higher compared with vitamin E (Miki 1991). With its superior antioxidant activity, astaxanthin has been used as a natural antioxidant in edible oil (Rao et al. 2007), nutraceuticals (Guerin et al. 2003), and cosmetics (Tominaga et al. 2012). Particularly, astaxanthin has shown great potential for promoting human health and in the prevention/treatment of various diseases (Table 5). Its efficiency has been proven in over 65 clinical studies and featured in over 300 peer-reviewed publications (Yamashita 2013). It should be noted that other carotenoids can act as a prooxidant under specific conditions such as high oxygen and partial pressure, while there is currently no information available regarding astaxanthin. Therefore, astaxanthin is considered as a high-value product due to its being increasingly marketed as a functional food ingredient with prices ranging between 3000 and $12,000 per kg (Lordan et al. 2011).

**Table 5:** Several beneficial effects of astaxanthin on promoting human health

Human health	Health benefits	References
Neurovascular protection	Decreases oxidation of red blood cells; decreases the chances of ischemic stroke; and improves memory and learning	Yook et al. (2015); Zhang et al. (2014a, b)
Eye fatigue relief	Reduces eye fatigue relieve in subjects suffering from visual display syndrome	Kajita et al. (2009); Kidd (2011); Nagaki et al. (2006); Serrano and Narducci (2014); Seya et al. (2009)
Immune system booster	Has an immunomodulating effect, strong immune system stimulator, anti-tumor, very effective for autoimmune conditions such as rheumatoid arthritis	Chew et al. (2010); Chew and Park (2004); Jyonouchi et al. (1995); Nir and Spiller (2002); Park et al. (2010)
Cardiovascular health	Improves blood lipid profiles, decreases blood pressure, offers protection from hypertension and stroke, reduces the consequences of a heart attack and vascular inflammation, reduces the area of infarction and the damage, reduces the area of infarction and the damage	Fassett and Coombes (2012); Fassett and Coombes (2011); Gross and Lockwood (2005); Guerin et al. (2003); Hussein et al. (2006); Hussein et al. (2005); Iwamoto et al. (2000); Miyawaki et al. (2008)
Liver health and metabolic syndrome	Improves blood lipids and increases adiponectin, prevents fatty liver disease, reduces the risk of atherosclerotic plaque, inhibits progression of fatty liver disease, restores insulin–glucose balance, increases fat burning, and decreases inflammatory markers	Kindlund and BioReal (2011); Kishimoto et al. (2016); Shen et al. (2014); Yilmaz et al. (2015)
Diabetes and Kidneys	Reduces glucose toxicity and kidney inflammation; improves pancreatic function, insulin resistance, and insulin sensitivity	Naito et al. (2004); Ni et al. (2015); Savini et al. (2013); Uchiyama et al. (2002)
Fertility	Improves sperm parameters and fertility	Comhaire et al. (2005); Donà et al. (2013); Mina et al. (2014)
Muscle resilience	Enhances power output, endurance, and recovery after exercise; prevents muscle damage and muscle atrophy	Earnest et al. (2011); Malmstena and Lignellb (2008); Yamashita (2011)
Capillary circulation	Improves blood flow and capillary integrity; reduces blood cell oxidation and risk of thrombosis	Kanazashi et al. (2013)
Anti-aging (skin cells)	Prevents UV-induced wrinkle formation, skin sagging, and age-spots; improves skin elasticity and skin dryness	Seki et al. (2001); Tominaga et al. (2012); Yamashita (2005)

## Industrially applicable techniques for efficient recovery of functional and bioactive nutraceuticals from lobster processing by-products

### Extraction of functional and nutritional proteins by isoelectric solubilization/precipitation and ultrasound-assisted extraction

Commercial lobster processing for fresh lobster meat, picked lobster meat, or canned lobster generates large amounts of lobster by-products containing residual meat, which is not frequently recovered by hand, or using mechanical equipment. This underutilization of the lobster results in a waste with highly valuable protein, which must be disposed of accordingly, frequently at a cost to the producers. Several possibly suitable techniques for the recovery of proteins from crustacean-, fish-, or meat-processing by-products have been considered. One of the most conventional methods used to transform fishery by-products into a marketable and consumer-friendly products is the use of endogenous or added proteolytic enzymes (Tong-Xun and Mou-Ming 2010; Venugopal and Shahidi 1995). However, the slow rate of hydrolysis, generation of short-chain peptides, loss of functionality of the native proteins, and the absence of homogeneous hydrolysates are other major limitations of this process (Kristinsson and Rasco 2000). Moreover, enzymes used often require an inactivation step and thus cannot be recycled for subsequent reactions leading to the rise of processing costs (Kristinsson and Rasco 2000). In addition, low yield, taste defects, and the overall economic feasibility are still major issues for using enzymatic hydrolysis on industrial scales. Other approaches, which have been explored, include the use of chemicals. Using acidic or alkaline solutions for degrading proteins into peptides of varying sizes is a nonselective and rapid method. However, the severe conditions (HCl 6 N, 118 °C, 18 h, or pH 12.5, 95 °C, 20 min) used in chemical hydrolysis can have negative consequences such as racemization, bitter taste, reduced nutritional quality, and poor functionality, resulting in products of lower value, i.e., fertilizer (Chobert et al. 1996).

A more promising technique is isoelectric solubilization and precipitation (ISP). The shifting in pH of the solutions used during this processing induces solubility of residual proteins while simultaneously separating of lipids and the inedible parts such as shells, membranes, bones, scales, and skin, not intended for human consumption (Gehring et al. 2011). Apart from its generating high yield of protein recovery, this process also produces high-quality proteins, which still maintain their functional properties and nutritional value (Chen et al. 2007b; Gigliotti et al. 2008; Nolsoe and Undeland 2009; Taskaya et al. 2009a; Taskaya et al. 2009b). Since the ISP process is simple and quick, it has been used for the recovery of fish proteins at both laboratory and pilot scales using batch mode (Choi and Park 2002; Kim et al. 2003; Kristinsson and Hultin 2003; Mireles Dewitt et al. 2002; Undeland et al. 2002). Furthermore, the presence of dioxin and polychlorinated biphenyls (PCBs), one of the most predominant bio-toxic compounds in fish protein, has been found to be reduced significantly in the ISP-recovered proteins (Marmon et al. 2009). When applied to other biological waste materials such as beef-processing by-products (Chen et al. 2007a; Mireles Dewitt et al. 2002) or chicken-processing by-products (Tahergorabi et al. 2011; Tahergorabi et al. 2012), high yields of protein recovery were still achieved. With its significant advantages over conventional methods, the ISP process could have a great potential for application on protein recovery from LPBs.

Recently, ultrasound-based extractions have been shown to be an effective technique for improving the rates of various extraction processes (Lebovka et al. 2011; Majid et al. 2015; Vilkhu et al. 2008). Ultrasound processing disrupts cells and creates microcavities in the tissue, which enhances the surface area and thus in the penetration of the solvent into the material, mass transfer, and improves protein release. The use of ultrasound in protein extraction from fish, meat, and beef by-products resulted in higher extraction yields with reduced processing time and solvent consumption compared with the use of ISP alone (Chemat and Khan 2011; Saleem et al. 2015; Vardanega et al. 2014; Vilkhu et al. 2008). Therefore, recovery of protein by ultrasound-assisted extraction has been scaled up to an industrial level due to their high economic feasibility (Álvarez and Tiwari 2015; Tu et al. 2015).

### Supercritical fluid extraction for recovery of rich-ω-3 lipids and astaxanthin

Solvent extraction is the most common method for lipid or astaxanthin extraction (Sindhu and Sherief 2011; Tsvetnenko et al. 1996). In this method, organic solvents including acetone, ethyl acetate, hexane, isopropanol, methanol, methyl ethyl ketone, ethanol, dichloromethane, dimethyl sulfoxide, or chloroform may be used for the extraction. Although some of these solvents can be used to extract lipids for food applications, others such as dichloromethane, dimethyl sulfoxide, and chloroform cannot be used due to their toxicity (FDA 2010). Regardless of the solvent used, there is increasing public awareness of the hazards related to the use of any organic solvents in the extraction of compounds for food or medical applications due to the possibility of solvent contamination in the final extracts. Apart from this, high demands for natural astaxanthin and bioactive lipid components such as ω-3 fatty acids, physterols, tocopherols, and tocotrineols have stimulated the search for green and sustainable extraction methods (Delgado Vargas and Paredes-Lopez 2003). One such approach that is being considered is the use of supercritical fluid extraction techniques.

In recent years, supercritical fluid extraction (SFE) has become an important technology for extracting high-quality lipids from fishery-processing by-products (Letisse et al. 2006; Rubio-Rodríguez et al. 2008). Furthermore, it is an effective separation technique in the production of nutraceutical supplements and functional foods (Parajó et al. 2008; Reverchon and De Marco 2006). The operational conditions of SFE are also favorable from an environmental and industrial processing viewpoint. SFE can extract bioactive nutraceuticals at moderate temperatures in a non-oxygen environment with very low lipid oxidation, selectively extracts low polar lipid compounds, and does not co-extract polar impurities such as some organic derivatives containing heavy metals (Rubio-Rodríguez et al. 2012).

Supercritical carbon dioxide (SC-CO_2_) extraction is presently being evaluated as a promising technology compared with conventional methods (López-Cervantes et al. 2006) due to its ability to extract the heat-sensitive, easily oxidized compounds (PUFAs, ω-3 fatty acids, and astaxanthin) without the use of toxic solvents. Moreover, CO_2_ is a generally recognized as safe (GRAS), relatively cheap, and easy to evaporate from the matrix and extracts (Mercadante 2008; Reverchon and De Marco 2006; Sahena et al. 2009). The high extraction yields achieved using this technique is attributable to the high diffusivity and solubility but low viscosity of SC-CO_2_. In contrast to the products extracted from the conventional methods, the SC-CO_2_ extracts are rich in nutraceuticals with high purity. SC-CO_2_ have been used to extract lipids and carotenoids from vegetables (Filho et al. 2008; Hardardottir and Kinsella 1988; Mendes et al. 1995; Silva et al. 2008) and animal matrices (Froning et al. 1990; Hardardottir and Kinsella 1988; Letisse et al. 2006; Tanaka and Ohkubo, 2003). The SC-CO_2_ extraction technique has also been studied for extraction of lipids and astaxanthin from crustacean-processing waste such as shrimp by-products (Charest et al. 2001; Felix-Valenzuela et al. 2001; Kamaguchi et al. 1986; Lopez et al. 2004). Recently, ω-3-rich lipids have been recovered with high yield (94%) from Rock lobster livers by SC-CO_2_ extraction (Nguyen et al. 2015).

### Microwave-intensified production of chitin and chitin derivatives

Chitin is found in lobster shells and is closely associated with proteins, minerals, and pigments, which need to be completely removed and separated from chitin during the extraction process. Although conventional methods removes nearly all these compounds from the shells, the use of strong chemicals and high temperatures during the process can cause deacetylation and depolymerisation leading to inconsistent physical properties of the extracted chitin (Jung et al. 2007; Kjartansson et al. 2006; Percot et al. 2003). In addition, under these harsh conditions, undesirable secondary reactions between amino acids and the alkaline medium as well as racemization occur rending the proteins and minerals unusable (Synowiecki and Al-Khateeb 2003). Moreover, the conventional method requires large volumes of water for washing steps, generating a huge amount of waste water (Wang and Chio 1998). To circumvent these issues, various biological processes have been employed for the production of chitin from crustacean shells such as fermentation or using commercially available enzymes (Giyose et al. 2010; Jung et al. 2007; Manni et al. 2010; Oh et al. 2007; Sini et al. 2007; Sorokulova et al. 2009; Xu et al. 2008). However, these proposed new methods require a longer production time (8–72 h), while their removal degrees (deproteinization, demineralization) are relatively low.

More recently, microwave has emerged as a promising nonconventional energy source for performing organic synthesis. Heat generated from microwave irradiation accelerates chemical reactions and enhances the rate of enzyme-catalyzed reactions so spectacularly that it cannot be explained by the effect of rapid heating alone (De La Hoz et al. 2005). Apart from thermal effects, microwave irradiation is accompanied with several nonthermal effects such as overheating, hot spots, selective heating, highly polarizing field, and mobility and diffusion. Microwave-assisted extraction has been proven to be an efficient technique for extracting small molecular weight from various biological samples due to its high ability to intensify processes, low usage of extraction chemicals, and shorter extraction time (Teo et al. 2013).

Microwave technology has been used as an environmentally friendly and cost-effective method for chitin production by assisting demineralization of deproteinized shrimp shells with lactic acid (Valdez-Pena et al. 2010). This process produced high yields of chitin with low residual minerals (0.2%); thus, demineralization of lobster shells by the microwave process was also optimized for chitin production obtaining promising results such as high degree of demineralization, low residues, and recovery of lobster minerals (Nguyen et al. 2017). The rate and yield of extraction can be further exploited by combining multiple processing approaches such as using microwave technology to increase the rate of enzymatic hydrolysis of lobster shells (Nguyen et al. 2016). Microwave irradiation has also been demonstrated as highly efficient in chemical deacetylation of chitin into chitosan. The degree of deacetylation in chitosan production within 5.5 min of microwave irradiation was as high as it was deacetylated at 121 °C, 15 psi for 4 h (Sahu et al. 2009). To generate chitosan with high solubility or desired functional properties, microwave irradiation has been extensively utilized for the chemical modification of chitosan (Ge and Luo 2005; Huacai et al. 2006; Liu et al. 2004). Particularly, the microwave has been demonstrated as a green and sustainable technology for degradation of chitin (Ajavakom et al. 2012; Roy et al. 2003) and chitosan (Garcıa et al. 2015; Li et al. 2012; Petit et al. 2015; Wasikiewicz and Yeates 2013) into low molecular weight chitosan or chito-oligomers in a wide range of applications.

## Conclusion

The global lobster processing industry produces a large amount of by-products with an estimated yield of 50,000 tons, which are currently being underutilized or discarded annually costing lobster companies in excess of $7.5 million/year for disposal. Finding alternative uses for this waste material could result in more environmentally friendly processes and ultimately result in an economic advantage for the lobster industry. This comprehensive review discusses the global availability of LPBs, the composition of the by-products, and the high-value compounds that may be extracted from them. It also discussed the key areas of applications—that these extracted compounds show potential to be used in—like water treatment, agriculture, food, nutraceutical, pharmaceutical products, and biomedicine. Furthermore, it addresses the limitations to current techniques used for the recovery of these valuable components and suggests emerging innovative techniques (i.e., microwave, ultrasonic, and supercritical fluid extraction) which may be more promising at the industrial scale. Although the potential value of such lobster ingredients is currently being ignored, several recent studies on biorefinery have shown that recovery of these valuable ingredients for value-added products is very promising due to their richness, highly commercial value, and numerous applications. Developing the simplified processes combined with using industrially applicable technologies for economic recovery of these valuable ingredients would be a practical solution for maximizing the utilization of these by-products. In this way, LPBs could be economically turned into a highly profitable source rather than a traditional pollution and costing source.

## Abbreviations

LPBs: lobster processing by-products
LSP: lobster shell protein
LPH: lobster protein hydrolysate
COS: chito-oligosaccharides
PUFAs: polyunsaturated fatty acids
SC-CO_2_: supercritical carbon dioxide
SFE: supercritical fluid extraction
EAA: essential amino acids
